# 10‐valent pneumococcal non‐typeable *Haemophilus influenzae* protein‐D conjugate vaccine (PHiD‐CV) induces memory B cell responses in healthy Kenyan toddlers

**DOI:** 10.1111/cei.12637

**Published:** 2015-06-10

**Authors:** D. M. Muema, E. W. Nduati, M. Uyoga, M. Bashraheil, J. A. G. Scott, L. L. Hammitt, B. C. Urban

**Affiliations:** ^1^ Pathogen, Vector and Host Biology DepartmentKEMRI‐Wellcome Trust Research ProgrammeCentre for Geographic Medicine‐CoastKilifiKenya; ^2^ Epidemiology and Demography ClusterKEMRI-Wellcome Trust Research ProgrammeCentre for Geographic Medicine-CoastKilifi, Kenya; ^3^ Department of Infectious Disease EpidemiologyLondon School of Hygiene and Tropical MedicineLondonUK; ^4^ Department of International HealthJohns Hopkins Bloomberg School of Public HealthBaltimoreMDUSA; ^5^ Department of ParasitologyLiverpool School of Tropical Medicine, Pembroke PlaceLiverpoolUK

**Keywords:** children, ELISPOT, memory B cells, PHiD‐CV, pneumococcal conjugate vaccines

## Abstract

Memory B cells are long‐lived and could contribute to persistence of humoral immunity by maintaining the plasma‐cell pool or making recall responses upon re‐exposure to an antigen. We determined the ability of a pneumococcal conjugate vaccine to induce anti‐pneumococcal memory B cells. Frequencies of memory B cells against pneumococcal capsular polysaccharides from serotypes 1, 6B, 14, 19F and 23F were determined by cultured B cell enzyme‐linked immunospot (ELISPOT) in 35 children aged 12–23 months who received pneumococcal non‐typeable *Haemophilus influenzae* protein‐D conjugate vaccine (PHiD‐CV). The relationships between plasma antibodies and memory B cell frequencies were also assessed. After two doses of PHiD‐CV, the proportion of subjects with detectable memory B cells against pneumococcal capsular polysaccharides increased significantly for serotypes 1 (3–45%; *P* < 0·01), 19F (21–66%; *P* < 0·01) and 23F (13–36%; *P* = 0·02), but not serotypes 6B (24–42%; *P* = 0·24) and 14 (21–40%; *P* = 0·06). Correlations between antibodies and memory B cells were weak. Carriage of serotype 19F at enrolment was associated with poor memory B cell responses against this serotype at subsequent time‐points (day 30: non‐carriers, 82% *versus* carriers, 0%, *P* < 0·01; day 210: non‐carriers, 72% *versus* carriers, 33%, *P* = 0·07). PHiD‐CV is capable of inducing memory B cells against some of the component pneumococcal capsular polysaccharides.

## Introduction

*Streptococcus pneumoniae* is a major cause of morbidity and mortality in the world, accounting for an estimated 478 000 deaths in children below 5 years of age in 2008 1. In countries where a pneumococcal conjugate vaccine has been introduced into the childhood immunization schedule, a dramatic reduction of the incidence of vaccine‐type invasive pneumococcal disease (IPD) among vaccinated children has been reported, as has indirect protection of unvaccinated individuals 2, 3, 4. *S. pneumoniae* is a leading cause of invasive bacterial disease in Kenyan children, and in 2011 the Kenyan Government introduced Synflorix^®^, the 10‐valent pneumococcal non‐typeable *Haemophilus influenzae* protein‐D conjugate vaccine (PHiD‐CV), into its childhood immunization programme 5, 6. The pneumococcal capsular polysaccharides in PHiD‐CV are conjugated to protein D of *H. influenzae* (serotypes 1, 4, 5, 6B, 7F, 9V, 14 and 23F), tetanus toxoid (serotype 18C) and diphtheria toxoid (serotype 19F).

The immunogenicity of pneumococcal vaccines has been assessed by measuring serum immunoglobulin (Ig)G [by enzyme‐linked immunosornebt assay (ELISA)] and opsonophagocytic activity (OPA). Studies in Europe, South America and Asia found comparable immunogenicity of PHiD‐CV and the 7‐valent pneumococcal conjugate vaccine (PCV7), even when co‐administered with other childhood vaccinations 7, 8, 9, 10, leading to licensure of PHiD‐CV in more than 120 countries. Antibody titres and OPA after vaccination wane over time, but increase markedly after booster vaccination, suggesting that the primary vaccination induces immunological memory 11.

Memory B cells form an important arm of humoral immunity, but unlike antibody responses these have not been investigated previously in the immune response to PHiD‐CV. For most antigens, after an initial antigenic challenge, both long‐lived plasma cells and memory B cells are generated 12. Long‐lived plasma cells constitutively secrete antibodies of a given specificity. Memory B cells are quiescent, but differentiate rapidly into short‐lived plasma cells upon secondary exposure to an antigen, thus boosting the concentrations of available circulating antibodies 13, 14. They have also been suggested to play a role in the maintenance of the plasma cell pool in absence of antigen, by being either activated polyclonally by pathogen‐associated molecular patterns or bystander T cell help 15. They can repopulate germinal centres and undergo further rounds of affinity maturation, resulting in an adapted population of memory and long‐lived plasma cells while maintaining the existing memory B cell population 14.

Memory B cells are maintained in the absence of cognate antigen, and this characteristic is thought to be responsible for the protection that is observed after waning of plasma antibodies to undetectable levels in individuals who are immunized against hepatitis B 16, 17. Indeed, they have been shown to protect against Japanese encephalitis in absence of plasma antibodies and CD8^+^ T cells in mice 18.

Following immunization with serogroup C meningococcal (MenC) glycoconjugate vaccine, the presence of circulating antibodies, as opposed to memory B cells, is the main determinant of protection from disease, probably because clinical disease develops within hours of infection before immunological recall responses are established 19. However, good memory responses have been associated with persistence of protective antibodies, suggesting that memory B cells could be indirectly important in determining the longevity of protection 20.

Assessment of the induction of memory B cells after vaccination provides important information about the durability of the immune response and could be a practical way of assessing the duration of protection. In this study, we aimed to determine whether vaccination with PHiD‐CV induced a serotype‐specific anti‐pneumococcal memory B cell response. We tested this in a study of Kenyan toddlers.

## Materials and methods

### Study participants

This analysis is a substudy of a double‐blind, randomized controlled trial that evaluated the immunogenicity, impact on nasopharyngeal carriage and reactogenicity of PHiD‐CV among 600 Kenyan children aged 12–59 months 21. In a randomly selected subset of 35 children aged 12–23 months who received PHiD‐CV at enrolment and 6 months later, the frequencies of antigen‐specific memory B cells were assessed on the day of enrolment before vaccination and 1 month after each dose of PHiD‐CV. Written informed consent was obtained from each participant's parent/guardian. The study protocol was reviewed and approved by the Kenya National Ethical Review Committee (SSC 1635) and the Oxford Tropical Ethical Review Committee (no. 54‐09).

### Cultured B cell enzyme‐linked immunospot (ELISPOT) for determination of frequencies of antigen‐specific memory B cells

Because of the limited amount of blood that could be obtained from the children, cellular assays were limited to serotypes 1, 6B, 14, 19F and 23F. These serotypes were chosen based on their contribution to IPD and nasopharyngeal carriage in the study setting 22, 23. Five ml blood were collected into sodium heparin tubes on the day of enrolment (just before the first dose), 30 days after enrolment (1 month after the first dose) and 210 days after enrolment (1 month after the second dose). The samples were then separated into plasma and cellular fractions by centrifugation. Peripheral blood mononuclear cells (PBMCs) were isolated by density gradient centrifugation, washed and frozen at 5 × 10^6^ cells/ml in freezing media comprising 10% dimethylsulphoxide (DMSO) (Sigma, St Louis, MO, USA) in fetal bovine serum (Invitrogen, Carlsbad, CA, USA).

Memory B cells against vaccine antigens were quantified using the method developed by Crotty *et al*. 24. Briefly, PBMCs were thawed, washed and cultured for 6 days in RPMI complete media (RPMI, 10% newborn bovine serum, 2 mM l‐glutamine, 100 units/ml penicillin, 0·1 mg/ml streptomycin, 10 mM Hepes, 0·5 mM β‐mercaptoethanol) containing 2·5 μg/ml cytosine–phosphate–guanine (CpG) oligodeoxynucleotides (ODN) 2006 (Hycult Biotech, Plymouth Meeting, PA, USA), 1 : 5000 dilution of *S. aureus* Cowan strain protein A (Sigma) and 0·083 μg/ml of pokeweed mitogen (Sigma) at 2 × 10^5^ cells per 200 μl in U‐bottomed 96‐well culture plates.

ELISPOT multi‐screen filter plates (Millipore, Darmstadt, Germany) were coated overnight at 4°C with either pneumococcal capsular polysaccharides for serotypes 1 (20 μg/ml), 6B (10 μg/ml), 14 (10 μg/ml), 19F (20 μg/ml) and 23F (20 μg/ml) (donation from GSK Biologicals, Rixensart, Belgium) in the presence of 10 μg/ml (1 and 14) or 20 μg/ml (6B, 19F and 23F) methylated human serum albumin (mHSA). Tetanus toxoid and diphtheria toxoid were coated at a concentration of 5 μg/ml, whereas goat anti‐human Igs (Caltag, Burlingame, CA, USA) were coated at 10 μg/ml. Negative control wells for determination of background responses were coated with mHSA alone at 10 μg/ml.

Thereafter, ELISPOT plates were washed three times with phosphate‐buffered saline (PBS) and blocked for at least 1 h with 10% newborn bovine serum (NBBS) in PBS. PBMCs harvested from the 6‐day cultures were seeded into the ELISPOT plates at either 200 and 2000 cells/well (total IgG‐secreting cells) or at 2 × 10^5^ cells/well (antigen‐specific IgG‐secreting cells) and incubated at 37°C in 5% carbon dioxide and 95% humidity overnight. Then, cells were discarded and plates washed four times with 0·25% Tween in PBS followed by a 5‐min soak with PBS. Wells were subsequently incubated with 50 μl/well of alkaline phosphatase‐conjugated donkey anti‐human IgG (Jackson ImmunoResearch Laboratories, West Grove, PA, USA) diluted with 10% NBBS in PBS at a dilution of 1 : 1000. After 4 h, the plates were washed five times with 0·25% Tween in PBS and then three times with distilled water. Spots were developed by adding 50 μl/well of alkaline phosphatase substrate (Bio‐Rad, Hercules, CA, USA). The reaction was stopped by washing with distilled water. The plates were left to dry then scanned using the ImmunoCapture software version 6·4 on a CTL Immunospot analyser. The spots were counted using pre‐established settings in ImmunoSpot version 5·0 software. For determination of frequencies of antigen‐specific memory B cells, background responses were subtracted from the antigen‐specific responses. A positive ELISPOT response was defined when the antigen‐specific response was at least five spots per 1 million PBMCs and at least twofold the background response, as defined elsewhere 25.

### Statistical analysis

Paired comparison of frequencies of memory B cells within individuals at different time‐points was performed using Wilcoxon's signed‐rank test. Other comparisons of frequencies of memory B cells between groups were performed using Wilcoxon's rank‐sum test. Correlations between frequencies of memory B cells and concentrations of plasma antibodies were determined using Spearman's correlation. Fisher's exact test was used to check for associations between memory B cell response and antibody response. McNemar's test of paired proportions was used to evaluate response within individuals at different time‐points. Differences were considered significant if *P* < 0·05. All statistical analyses were performed using stata version 13 (StataCorp, College Station, TX, USA).

## Results

### Participant characteristics

The characteristics of the 35 children in the memory B cell substudy are shown in Table 1. Subjects’ median age was 20 months and 54% were female. All children had received at least one dose of pentavalent immunization (against diphtheria, tetanus, pertussis, *H. influenzae* type b and hepatitis B) in the first year of life, while 91.4% had received all three recommended doses. Nasopharyngeal carriage was assessed in the parent study. Among these substudy participants, there was a decline in overall pneumococcal carriage from 88·6% (31 of 35) at day 0 to 71·4% (25 of 35) at day 180. There was also a reduction in carriage of vaccine‐type pneumococci from 42·9% (15 of 35) at day 0 to 25·7% (nine of 35) at day 180. The carriage rate of non‐vaccine serotypes remained unchanged at 45·7% (16 of 35). These findings are consistent with the patterns observed in the parent study 21.

**Table 1:** Characteristics of the children who participated in the study

Characteristic	Value
Total number	35
Median age (IQR) in months	20 (16–22)
Females (%)	19 (54%)
Median children in household (range)	3 (1–6)
Smokers in household (%)	11 (31%)
Received 3 doses of pentavalent vaccine	91.4%
Received at least 1 dose of pentavalent vaccine	100.0%

IQR = interquartile range.

### Antigen‐specific immune responses

IgG levels against the vaccine serotypes were determined in the parent study 21. Among substudy participants, PHiD‐CV induced increases in the proportions of participants attaining the putative protective threshold of 0·35 μg/ml of anti‐capsular IgG against all tested pneumococcal capsular polysaccharides after the first and second doses, consistent with findings of the parent study (Table 2) 26, 27. To a lesser extent, PHiD‐CV also induced increases in proportions of participants that had detectable memory B cells against some of the pneumococcal antigens. The proportions of children with memory B cells against serotypes 1, 19F and 23F at day 210 (after the two doses of PHiD‐CV) and serotypes 1 and 19F at day 30 (after the first dose of PHiD‐CV) were significantly higher when compared to respective proportions at day 0. PHiD‐CV did not alter significantly the proportions of participants who had detectable memory B cells against serotypes 6B and 14 (Table 2).

**Table 2:** Percentages of participants with serotype‐specific immunoglobulin (Ig)G responses and cultured B cell enzyme‐linked immunospot (ELISPOT) responses

	IgG ≥ 0·35 μg/ml	*P*‐value	ELISPOT response	*P*‐value
Day 0				
Serotype 1	3%		3%	
Serotype 6B	29%		24%	
Serotype 14	26%		21%	
Serotype 19F	23%		21%	
Serotype 23F	9%		13%	
Tetanus toxoid			81%	
Diphtheria toxoid			62%	
Day 30				
Serotype 1	91%	<0·01	57%	<0·01
Serotype 6B	66%	<0·01	29%	0·25
Serotype 14	83%	<0·01	36%	0·06
Serotype 19F	77%	<0·01	61%	0·02
Serotype 23F	60%	<0·01	24%	0·25
Tetanus toxoid			86%	1·00
Diphtheria toxoid			100%	
Day 210				
Serotype 1	97%	<0·01	45%	<0·01
Serotype 6B	97%	<0·01	42%	0·24
Serotype 14	100%	N/A	40%	0·06
Serotype 19F	91%	<0·01	66%	<0·01
Serotype 23F	91%	<0·01	36%	0·02
Tetanus toxoid			94%	0·22
Diphtheria toxoid			83%	0.38

Statistical test use: McNemar's test of paired proportions for day 0 *versus* day 30 and day 0 *versus* day 210. Differences were considered significant if *P* < 0·05.

**Figure 1:**
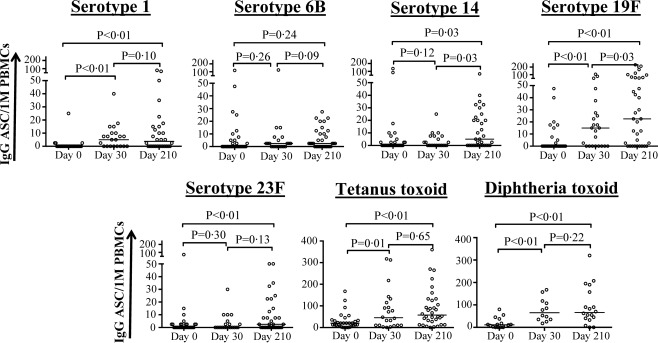
Induction of memory B cells by pneumococcal non‐typeable *Haemophilus influenzae* protein‐D conjugate vaccine (PHiD‐CV). Frequencies of antigen‐specific memory B cells against various pneumococcal capsular polysaccharides, tetanus toxoid (TT) and diphtheria toxoid (DT) are shown in scatter dot‐plots. Horizontal line represents median. Statistical test used: Wilcoxon's signed‐rank test. *P*‐values < 0·05 were considered significant. Immunoglobulin (Ig)G ASC/1M PBMCs, IgG antibody‐secreting cells per 1 million peripheral blood mononuclear cells (PBMCs).

Frequencies of antigen‐specific memory B cells at baseline and after the first and second doses of PHiD‐CV are shown in Fig. 1. The highest baseline and vaccine‐induced frequencies of memory B cells were observed for serotype 19F. After each dose of PHiD‐CV, there was a significant increase in the median frequency of memory B cells against serotype 19F [day 0: 0 (interquartile range IQR = 0–4); day 30: 15 (IQR = 0–28); day 210: 23 (IQR = 0–61)]. For serotype 14, the median frequency of memory B cells did not change significantly after the first dose of PHiD‐CV, but increased significantly after the second dose of PHiD‐CV [day 0: 0 (IQR = 0–3); day 30: 0 (IQR) = 0–8; day 210: 5 (IQR = 0–21)]. A similar pattern was observed for serotype 23F [day 0: 0 (IQR = 0–3); day 30: 0 (IQR = 0–4); day 210: 3 (IQR = 0–14)]. Median frequency of memory B cells against serotype 1 was higher after the first and second doses of PHiD‐CV when compared with baseline [day 0: 0 (IQR) = 0–0); day 30: 5 (IQR = 0–10); day 210: 4 (IQR = 0–15)]. There was no significant increase in the median frequency of memory B cells against serotype 6B after either dose of PHiD‐CV [day 0: 0 (IQR = 0–0); day 30: 3 (IQR = 0–6); day 210: 3 (IQR = 0–12)]. Notably, these kinetics of memory B cells differed from those of plasma antibodies, suggesting independence in the generation of the two compartments. There were significant increases in plasma antibodies against all assessed serotypes after each dose of PHiD‐CV, with the highest increases being observed after the first dose 21.

In PHiD‐CV, the capsular polysaccharides for serotypes 18C and 19F are conjugated to tetanus toxoid and diphtheria toxoid, respectively. PHiD‐CV would therefore be expected to boost pre‐existing responses against diphtheria toxoid and tetanus toxoid, components of diphtheria, tetanus and acellular pertussis immunization (DTaP) administered to children in the first year of life. The children in this study also received a booster dose of DTaP vaccine on day 60. We therefore determined the effect of the study vaccination regimen on the memory B cell responses against tetanus toxoid and diphtheria toxoid. Administration of the first dose of PHiD‐CV induced a significant increase in the median frequency of memory B cells against tetanus toxoid as measured on day 30 [day 0: 20 (IQR = 5–33) *versus* day 30: 45 (IQR = 10–119)]. However, administration of a booster dose of DTaP on day 60 or a second dose of PHiD‐CV on day 180 did not further change the frequencies of memory B cells against tetanus toxoid as measured on day 210 [day 30: 45 (IQR = 10–119) *versus* day 210: 58 (IQR = 24–127)]. A similar effect was observed with regard to frequencies of memory B cells against diphtheria toxoid [day 0: 11 (IQR = 1–33); day 30: 65 (IQR = 35–115); day 210: 66 (IQR = 38–166)] (Fig. 1). Notably, the vaccination schedule did not alter significantly the proportions of participants that had detectable memory B cells against tetanus toxoid and diphtheria toxoid, probably because the participants had high pre‐existing responses.

When the participants were stratified into two age groups based on a cut‐off of 18 months of age, 13 participants were in the younger age group (median age = 16 months) whereas 22 were in the older age group (median age = 22 months). There were no differences in proportions of memory B cell responders for any serotype when the older age group was compared with the younger age group, with the exception of serotype 6B on day 30, whereby the younger age group had a higher proportion of responders (60 *versus* 0%, *P* < 0·01).

### Relationship between plasma IgG and memory B cells against pneumococcal capsular polysaccharides

Upon antigenic challenge, usually both quiescent memory B cells and active antibody‐secreting long‐lived plasma cells are generated 12. Here, we checked for associations between plasma antibodies and memory B cell responses against the pneumococcal antigens. Due to the limited sample that could be obtained from the participants and the sampling times that were outside the period when plasma cells would be expected in peripheral circulation, we could not directly enumerate plasma cells 28. We chose to use previously measured plasma antibody concentrations as a proxy indicator of the number of plasma cells generated.

**Figure 2:**
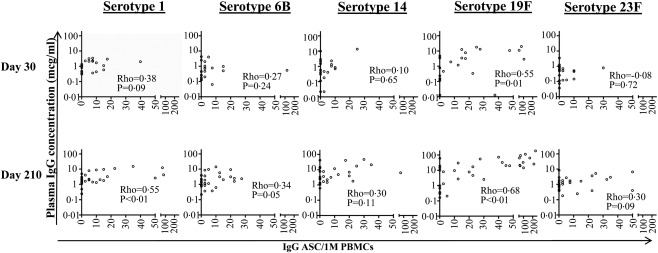
Relationship between plasma antibodies and memory B cells. Correlations between plasma antibodies and frequencies of memory B cells against various pneumococcal capsular polysaccharides at different time‐points are shown (top panel –day 30; bottom panel –day 210). Statistical test: Spearman's correlations. *P*‐values < 0·05 were considered significant. Immunoglobulin (Ig)G antibody‐secreting cells (ASC)/1 M peripheral blood mononuclear cells (PBMCs), IgG antibody‐secreting cells per 1 million PBMCs.

**Figure 3:**
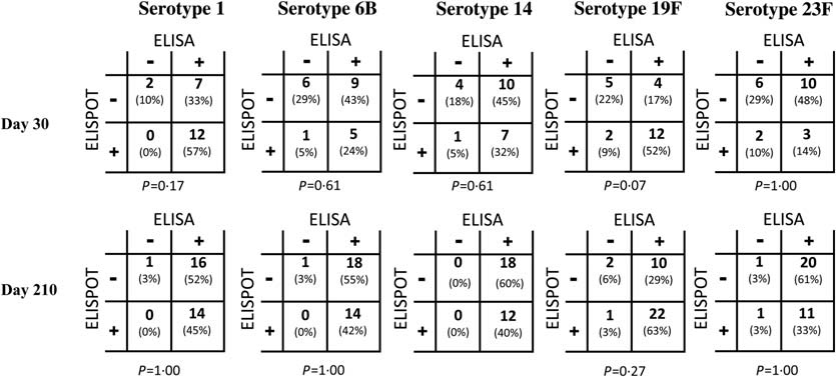
Relationship between plasma antibodies and memory B cells. Number and proportion of participants who attained plasma immunoglobulin (Ig)G ≥ 0·35 μg/ml and/or had detectable memory B cells against various pneumococcal capsular polysaccharides at different time‐points are shown (top panel: day 30; bottom panel: day 210). Statistical test: Fisher's exact test. *P*‐values < 0·05 were considered significant. Enzyme‐linked immunosorbent assay (ELISA)^+^: plasma IgG ≥ 0·35 μg/ml. Enzyme‐linked immunospot (ELISPOT)^+^: antigen‐specific response ≥ 2 times of background response and ≥ 5 antibody‐secreting cells (ASC)/1 M peripheral blood mononuclear cells (PBMCs).

Plasma antibody concentrations and the frequencies of antigen‐specific memory B cells correlated for serotype 19F on day 30 (after the first dose of PHiD‐CV) and serotypes 1, 6B and 19F on day 210 (after the second dose of PHiD‐CV) (Fig. 2). However, when the categorical data on responders *versus* non‐responders were considered, there were no significant associations between antibody responsiveness and memory B cell responsiveness, suggesting minimal association between plasma antibodies and memory B cells against pneumococcal capsular polysaccharides upon vaccination with PHiD‐CV (Fig. 3).

### Relationship between baseline pneumococcal carriage and anti‐pneumococcal memory B cells after vaccination

**Figure 4:**
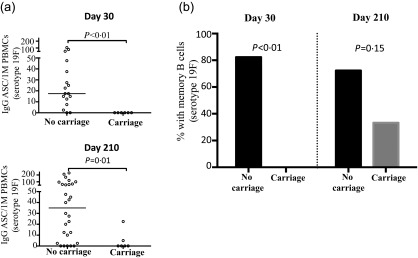
Relationship between baseline pneumococcal carriage and memory B cell responses after vaccination with pneumococcal non‐typeable *Haemophilus influenzae* protein‐D conjugate vaccine (PHiD‐CV). (a) Frequencies of antigen‐specific memory B cells against serotype 19F by baseline pneumococcal carriage of serotype 19F. Horizontal line represents median. Statistical test used: Wilcoxon's rank‐sum test. *P*‐values < 0·05 were considered significant. Immunoglobulin (Ig)G antibody‐secreting cells (ASC)/1 M peripheral blood mononuclear cells (PBMCs). (b) Proportions of study participants who had detectable memory B cells against serotype 19F on days 30 and 210 after stratifying on the basis of baseline serotype 19F carriage. Statistical test used: Fisher's exact test. *P*‐values < 0·05 were considered significant.

Some studies have reported serotype‐specific antibody hypo‐responsiveness to pneumococcal antigens in individuals that have nasopharyngeal carriage of the respective serotypes at the time of vaccination 29, 30. To determine if carriage at baseline affected the memory B cell response to vaccination, we stratified children on the basis of their carriage status on the day of enrolment and compared the subsequent responses between the two groups. This analysis was limited to serotype 19F, the only serotype that was carried by more than two participants at baseline, had detectable memory B cells in at least either carriers or non‐carriers group and had data on frequencies of memory B cells for the carrying participants in subsequent time‐points. Baseline carriage of serotype 19F was associated with a lower proportion of responders and lower frequencies of memory B cells against the serotype upon vaccination with PHiD‐CV: when compared with children who did not have carriage for serotype 19F at day 0, the six children that had carriage had significantly lower frequencies of memory B cells against serotype 19F on day 30 [carriers: 0 (IQR = 0–0); non‐carriers: 18 (IQR = 10–43)] and day 210 [carriers: 0 (IQR = 0–9); non‐carriers: 35 (IQR = 4–66)] (Fig. 4a). On day 30, baseline serotype 19F carriage was associated with a significantly lower proportion of participants with detectable memory B cells against serotype 19F (non‐carriers: 82% *versus* carriers: 0%, *P* < 0·01), but a significant difference was not observed on day 210 (non‐carriers: 72% *versus* carriers: 33%, *P* = 0·07) (Fig. 4b).

## Discussion

In this study, we report that vaccination of young children with PHiD‐CV induces memory B cells against select pneumococcal capsular polysaccharides contained in the vaccine and boosts the frequency of memory B cells against two carrier antigens, diphtheria toxoid and tetanus toxoid. This suggests that vaccination may provide long‐lived protection against vaccine‐type IPD.

Our findings are consistent with serological studies showing that booster vaccination after primary vaccination with PHiD‐CV induces higher antibody responses than primary vaccination alone, suggesting that PHiD‐CV induces B cell memory upon primary vaccination 11, 31, 32. Indeed, Clutterbuck *et al*. demonstrated that infants are capable of generating serotype specific memory B cells upon vaccination with the heptavalent pneumococcal conjugate vaccine 33. Memory B cell responses to three of the serotypes we studied – 1, 6B and 19F – were also detected by van Westen *et al*. in Dutch children vaccinated with PCV13 or PHiD‐CV at 2, 3, 4 and 11 months of age 34. However, the absence of significant vaccine‐induced memory B cell responses against serotypes 6B and 14 in our study, as opposed to the other serotypes in our study and serotype 6B in the van Westen study, suggests that the induction of memory B cells may vary by serotype and geographical location, despite consistency in induction of plasma antibodies. Alternatively, the increases in vaccine‐induced memory B cell responses against serotypes 6B and 14 may have reached statistical significance if the sample size had been larger. These differences are unlikely to undermine the immediate protection against the respective serotypes, but it would be important to determine if they result in varying long‐term protection from disease.

The presence of some memory B cell responses against all serotypes at baseline in some of the children suggested that natural exposure or previous disease may have induced the formation of memory B cells against the capsular polysaccharides. In agreement, antibody responses against pneumococcal capsular polysaccharides increase upon natural exposure and are thought to contribute to the age‐dependent naturally acquired immunity to pneumococcal disease 35. However, we do not expect natural exposure to have contributed significantly to the increase in anti‐pneumococcal responses during the study. First, nasopharyngeal carriage of vaccine‐type pneumococci declined during the study period, reducing the possibility of participants gaining natural antigen challenge. Furthermore, the older age group in this study did not have higher proportions of responders, suggesting that the natural acquisition of responses could be slow and would not have a significant impact on increase in immune responses during the study period.

The prevalence and magnitude of baseline and vaccine‐induced memory B cell responses against the different serotypes differed. Differences in sensitivity of the ELISPOT assay for different serotypes are unlikely to explain the variation, as PBMCs were cultured in the same culture conditions regardless of the ELISPOT‐coating antigen. However, pre‐existing responses due to natural exposure, nature of carrier protein and nature of the polysaccharide itself could be important in determining responses to each serotype. For instance, responses against serotype 19F were much higher than those against any of the other four serotypes tested, probably because serotype 19F is carried more commonly in the nasopharynx of children in the studied community 22. Memory B cell responses against serotype 19F after vaccination with PHiD‐CV were also stronger than those of any other serotype measured. The high baseline memory B cell responses against serotype 19F suggest that children had already been primed against serotype 19F, thereby leading to higher vaccine‐induced responses to the serotype compared to other serotypes. In addition, as serotype 19F is conjugated to diphtheria toxoid, an antigen that children are exposed to through childhood pentavalent vaccination series, the amount of prevaccination memory T cell help available to serotype 19F‐specific B cells may be higher than for other antigens. Similarly, responses against tetanus toxoid and diphtheria toxoid were considerably higher than those against any of the pneumococcal polysaccharides, probably because the children had received three doses of the pentavalent vaccine in childhood in addition to boosting by PHiD‐CV and DTaP doses that they received during the study. Furthermore, tetanus toxoid and diphtheria toxoid, being proteins, would be expected to elicit T cell help more readily when compared with polysaccharides, hence leading to stronger B cell responses 36. Similar differences between memory B cells against tetanus toxoid, diphtheria toxoid and pneumococcal capsular polysaccharides have been reported in children elsewhere 33. It would have been interesting to determine if the vaccine induced similar memory B cell responses against protein D of *H. influenzae*. This antigen was not available; however, we would expect responses to be similar to those induced against the other conjugating proteins.

Both memory B cells and plasma cells are generated as part of B cell responses. However, even though the numbers of memory B cells and plasma cells generated are often correlated, the processes of generating the two B cell subsets are independent 37. This provides room for discordance between the two subsets and raises the question as to whether measuring circulating antibodies alone provides a comprehensive picture of the B cell response, especially the predicted longevity of B cell responses. The differences in kinetics and absence of correlations between memory B cells and plasma antibodies in this study are in line with the independence between the two B cell subsets and emphasize the importance of measuring memory B cells directly when determining the induction of memory by PHiD‐CV.

We observed lower memory B cell responses against serotype 19F in children who were carrying serotype 19F in the nasopharynx at baseline. An inverse relationship between carriage and serotype‐specific IgG levels upon vaccination with pneumococcal vaccines has been postulated to be related to serotype‐specific exhaustion of relevant B cell clones that could blunt the antibody response against the colonizing serotype 29, 30. Alternatively, immunological priming through natural exposure could have contributed to the observed effect. Pneumococcal nasal challenge without resultant carriage has been shown to induce mucosal IgG against protein antigens, implying that priming and boosting of immune responses can occur in the absence of active carriage as long as an individual is exposed to the bacteria 38. Such priming can prevent nasopharyngeal carriage upon subsequent exposure. In our study, it is probable that both carriers and non‐carriers had previously experienced natural exposure, with the non‐carriers eliciting better priming of systemic B cell responses while also making better mucosal responses that would prevent carriage. Such priming in the children without nasopharyngeal carriage would explain their better responses to the vaccine when compared with children who had baseline carriage.

This study shows that PHiD‐CV is capable of inducing memory B cells against pneumococcal capsular polysaccharides in children aged 12–24 months, which could contribute to a long‐lasting protective effect against pneumococcal disease. It would be important to assess if frequencies of memory B cells correlate with persistence of protection.

## Disclosure

The authors have the following interests: J. A. G. S. has received research funding from GlaxoSmithKline Biologicals and support for travel/accommodation at a scientific meeting sponsored by Merck. L. L. H. has received research funding from GlaxoSmithKline Biologicals and Pfizer, Inc. There are no patents, products in development or marketed products to declare.

## Author contributions

B. C. U., L. L. H. and J. A. G. S. conceived and designed the experiments, D. M. M., M. U. and M. B. performed the experiments, D. M. M., E. W. N., B. C. U. and L. L. H analysed the data and D. M. M., E. W. N., L. L. H. and B. C. U. wrote the paper. D. M. M. is funded by a strategic award from the Wellcome Trust (no. 084538). E. W. N., J. A. G. S. and B. C. U. are funded by fellowships from the Wellcome Trust (nos 095068, 098532 and 079082, respectively). This was an investigator‐initiated study funded by GlaxoSmithKline Biologicals.
